# Lung Stereotactic Body Radiotherapy (SBRT) on Metastatic Sarcomas: A Retrospective Study at a Quaternary Center

**DOI:** 10.7759/cureus.93346

**Published:** 2025-09-27

**Authors:** Pedro Ferreira, Susana Esteves, João G Fonseca, Eduardo Netto

**Affiliations:** 1 Radiation Oncology, Instituto Português de Oncologia de Lisboa Francisco Gentil, Lisbon, PRT; 2 Clinical Investigation Unit, Instituto Português de Oncologia de Lisboa Francisco Gentil, Lisbon, PRT; 3 Comprehensive Health Research Center, NOVA Medical School, Universidade NOVA de Lisboa, Lisbon, PRT

**Keywords:** lung, oligo-metastases, sarcomas, sbrt, systemic therapy-free survival, local control

## Abstract

Introduction and purpose: Lung metastases are common in sarcoma patients, often necessitating systemic therapy or local therapies. Stereotactic body radiotherapy (SBRT) offers a targeted approach, achieving low local failure (LF) and delaying systemic therapy. This study evaluates the outcomes of SBRT for sarcoma lung metastases, focusing on LF, systemic therapy-free survival (STFS), and overall survival (OS).

Methods and materials: Retrospective study of patients with metastatic pulmonary lesions treated with SBRT between September 2015 and December 2022. STFS was defined as the time from the end of the first RT course to the start of any systemic therapy, if no previous systemic therapy was given or if a pause of at least one month was achieved with RT. STFS was depicted as a Kaplan-Meier curve. LF was analyzed using a competing risks framework. The response assessment was evaluated based on pre- and post-treatment chest CT or PET-CT. Toxicities were classified according to the Common Terminology Criteria for Adverse Events (CTCAE), version 5.0.

Results: A total of 17 patients and 53 lesions were treated in 31 courses of treatment. Median follow-up was 96 months. Median number of lesions treated per treatment course was 2 (range: 1-4). In 94.1% of the treatment courses, the target was the only active site of active disease. In 79.2% of the lesions treated, the biologically effective dose (BED) was equal to or superior to 100Gy with a median BED of treated lesions of 105.6Gy (range: 67.2-180Gy). No SBRT-related toxicities were reported. LF rates at one-, two-, and three-years were 32.0%, 45.3% and 47.2%, respectively. Median STFS was 26 months. STFS rates at one- and two-years were 62% (CI95% 40-95) and 54% (CI95% 33-89), respectively. Median OS was 34 months. OS rates at one- and two-years were both 71% (CI95% 53.3-85.6).

Conclusion: SBRT is a promising strategy for managing lung metastases in sarcoma, offering acceptable local control (LC) rates and significant systemic therapy-free survival without accrued toxicities. Further research should focus on optimizing dose schedules and patient selection to enhance outcomes.

## Introduction

Bone and soft tissue sarcomas are a rare group of cancers with enormous histological heterogeneity [1]. These tumors have a high tendency for distant metastasis, with the lung being the most common site [2-5].

In patients with pulmonary metastases in the context of oligometastatic disease, local therapies such as surgery or stereotactic body radiotherapy (SBRT) can play an important role in improving overall survival (OS) and delaying the need for systemic therapy [6].

Metastasectomy for pulmonary metastases has been described since 1882; multiple factors can influence intervention in this context: the number, location, and size of lesions, symptoms and performance status, comorbidities, and patient preference [7]. It is also important to consider that patients on therapeutic systemic therapy may require interruption to reduce surgical complications, thus increasing the risk of progression during the time of systemic therapy pause.

SBRT is a highly conformal technique that allows radiotherapy to be delivered with ablative doses of radiation in a limited number of fractions. Regarding SBRT in oligometastatic patients with pulmonary metastasis, it has been proven that a radical attitude at the local level can improve clinical results, particularly regarding local control (LC) with prolonged OS [8,9].

Radiotherapy in the context of sarcomas is typically considered a second-line alternative, considering the radioresistant nature of these neoplasms. However, with the advent of SBRT and the ability to deliver treatment at higher biologic equivalent dose levels, this paradigm is evolving. Several studies have already demonstrated the capacity for LC of metastatic lung sarcoma lesions, with good efficacy and minimal toxicity. In addition to the good LC, this radical local therapy strategy can also allow pauses in systemic therapy or postpone the start of it, thus providing a strategic advantage in managing the disease.

We propose to investigate the oncological outcomes of lung SBRT on metastatic sarcomas in a single institution, with a focus on local control and systemic therapy-free survival (STFS).

## Materials and methods

Population

We queried radiation treatment courses in our department's internal database and cross-linked with the institution’s medical records for patients with metastatic pulmonary lesions from sarcoma treated with SBRT between September 2015 and September 2022. SBRT was defined as image-guided radiotherapy delivered in between one and 12 fractions with a biologically effective dose (BED) higher than 48. The BED was determined assuming an alpha/beta ratio of 10 Gy for pulmonary metastases. Inclusion criteria were 18 or more years old, a histologic diagnosis of high-grade sarcoma, and pulmonary lesions in a context of oligometastatic disease. Patients outside this time frame or treated with palliative radiotherapy were excluded.

Treatment planning

All patients were treated with a 4D-CT simulation, 2-mm-thick slices, with vacuum cushion immobilization and both arms raised above the head using an arm rest. The treatment volumes were defined as follows: the gross tumour volume (GTV) was contoured on five different respiratory phases, namely the end-inspiratory, the end-expiratory phases and three intermediate phases, no margin for microscopic disease extension was used; the internal target volume (ITV) was generated by merging GTVs from all phases; the planning target volume (PTV) was generated by a geometrical margin of 0.5 cm around the ITV. The treatment was delivered with free breathing and daily megavoltage cone beam CT (MV-CBCT)-based guidance to verify the treatment position.

End-points definition

Local failure (LF) was calculated as the time from the end of radiotherapy (RT) to progression of the treated lesion. To be considered progression, an increase of 20% in tumor control (TC) was considered. The response assessment was evaluated based on pre-and post-treatment chest CT or PET-CT. Systemic therapy-free survival (STFS) was defined as the time from the end of the first RT cycle to the start of any systemic therapy if no previous systemic therapy was given or if a pause of at least one month was achieved with RT (to exclude the pauses in systemic therapy only due to the RT treatment). Overall survival (OS) was defined as the time between the end of the first RT cycle and the date of death from any cause. Toxicities were classified according to Common Terminology Criteria for Adverse Events (CTCAE), version 5.0 [10].

Statistical analysis

Patient characteristics were summarized with the use of descriptive statistics. Median follow-up was calculated using a reverse Kaplan-Meier. OS and STFS were estimated using a Kaplan-Meier curve. Cumulative incidence of LF was calculated using a competing risks framework where death without local failure was a competing event. We did not adjust for clustering due to multiple lesions from the same patient.

## Results

Patient and treatment characteristics

A total of 17 patients were identified, 10 (58.8%) were men, the median age was 50 years (range: 18-75 years), and all had an Eastern Cooperative Oncology Group Performance Status (ECOG-PS) of 0 or 1 (Table 1). All patients were discussed at a multidisciplinary board when deciding on SBRT over other treatments, and the diagnostic image was a thorax CT. The most common disease histology was synovial sarcoma (n = 5, 29.4%) and osteosarcoma (n = 4, 23.5%). In almost all patients, the intrathoracic target was the only active site of active disease (n = 16; 94.1%), and a majority had received prior systemic therapy (n = 11; 64.7%). The median follow-up was 96 months.

**Table 1:** Baseline patient characteristics * Other histologies include (all n=1): fibromyxoid sarcoma, epithelioid sarcoma, chondrosarcoma, fusocellular/sclerosing rhabdomyosarcoma, alveolar soft tissue sarcoma, undifferentiated sarcoma ** At the time of the first course of SBRT ECOG-PS: Eastern Cooperative Oncology Group Performance Status; SBRT: stereotactic body radiotherapy

Patient characteristic	N = 17; N (%)
Median age in years (range)	50 (18-75)
Gender	
Male	10 (58.8)
Female	7 (41.2)
ECOG-PS 0-1	
Yes	17 (100)
No	0 (0)
Histology	
Synovial sarcoma	5 (29.4)
Leiomyosarcoma	2 (11.7)
Osteosarcoma	4 (23.5)
Other*	6 (35.3)
Intrathoracic only disease**	
Yes	16 (94.1)
No	1 (5.9)
Prior systemic therapy**	
Yes	11 (64.7)
No	6 (35.3)

The patients were treated in 31 treatment courses, and eight patients were treated with more than one course. The median time between courses in patients treated more than once was eight months (range: 2-25 months). The median number of lesions treated per treatment course was two (range: 1-4 lesions). In 20 (64.7%) treatment courses, synchronous SBRT was delivered to more than one lung metastasis, but in most treatment courses, only one or two lesions were targeted (n= 24; 82.4%) (Table 2).

**Table 2:** Treatment-related characteristics per radiotherapy course

Treatment characteristics per course	N = 31; N (%)
Median number of treated lesions (range)	2 (1-4)
Number of treated lesions	
1	11 (35.3)
2	13 (47.1)
3 or more	7 (17.6)

A total of 53 lesions were treated with a median BED10 of 105.6Gy (range: 67.2-180Gy) (Table 3). The most common dose and fractionation was 48Gy in four fractions, and in almost all treatments the BED was equal to or superior to 100Gy (n = 42; 79.2%). The most common fractionation with a BED inferior to 100Gy was 48Gy in 12 fractions, and the last recorded treatment with a BED within that range was in September 2020.

**Table 3:** Treatment-related characteristics of metastases or local recurrences treated by SBRT in the lung (per lesion) * Other fractionations include (all n=1): 45 Gy/3 fractions, 48 Gy/3 fractions, 56 Gy/8 fractions, 60 Gy/3 fractions, 60 Gy/8 fractions SBRT: stereotactic body radiotherapy

Treatment characteristics per lesion	N = 53; N (%)
Dose fractionation	
45 Gy/5 fractions	3 (5.7)
48 Gy/4 fractions	30 (56.6)
48 Gy/12 fractions	7 (13.2)
50 Gy/5 fractions	8 (15.1)
Other*	5 (9.4)
BED10 > 100Gy	
Yes	42 (79.2)
No	11 (20.8)

Treatment outcomes

**Figure 1:**
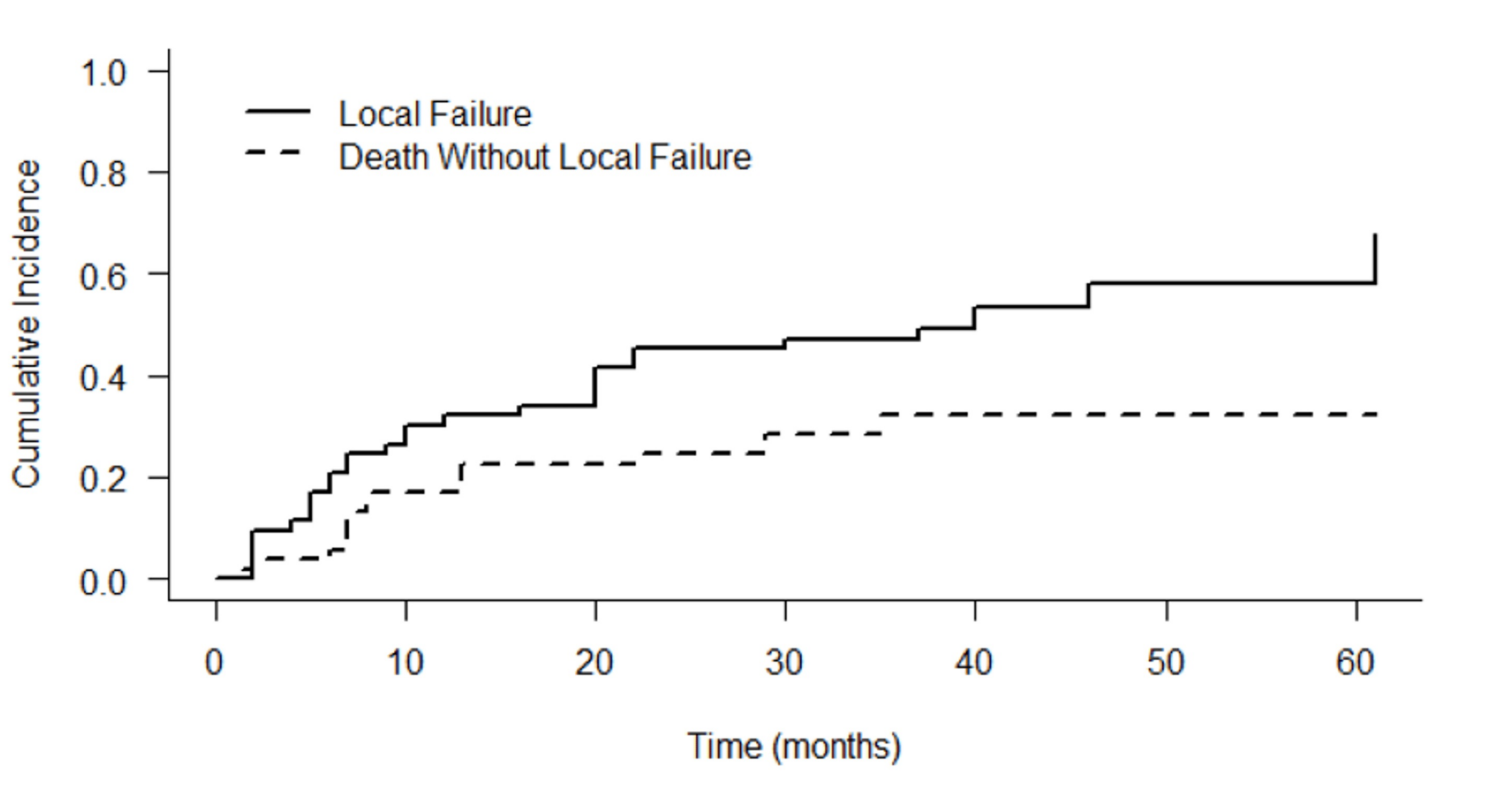
Cumulative incidence of local failure and death without local failure Cumulative incidence of local failure (solid line) and death without local failure (dashed line) over time (months) following lung SBRT for sarcoma metastases SBRT: stereotactic body radiotherapy

Cumulative incidence of local failure, with death as a competing risk, at one-, two-, and three-year was 32.0%, 45.3% and 47.2% respectively (Figure 1). The cumulative incidence of death without local failure was 17.0% at one year and 24.5% at two years. Death without local failure was consistently lower; therefore, local failure was the predominant event. All local failures were diagnosed radiographically.

**Figure 2:**
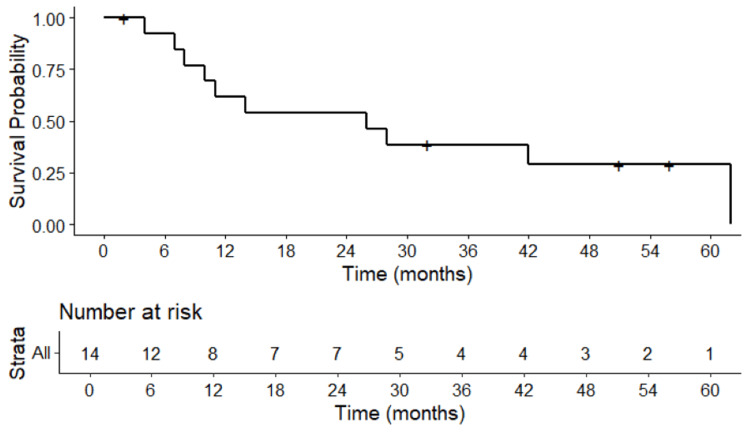
Kaplan-Meier survival curve showing the probability of systemic therapy-free survival over time (months) following lung SBRT for sarcoma metastases SBRT: stereotactic body radiotherapy

**Figure 3:**
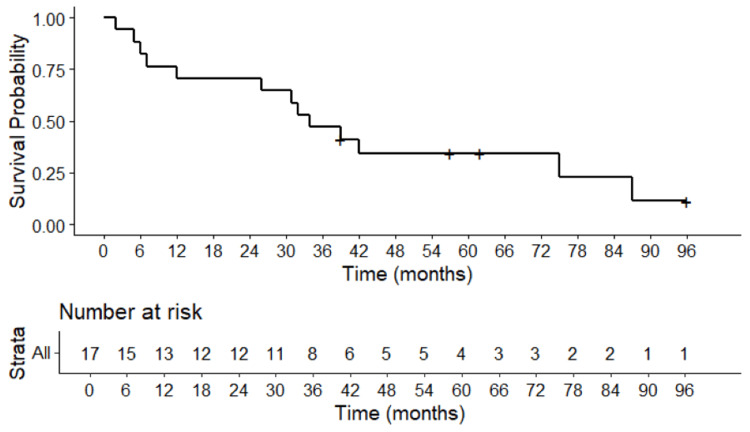
Kaplan-Meier survival curve showing the probability of survival over time (months) following lung SBRT for sarcoma metastases SBRT: stereotactic body radiotherapy

The median STFS was 26 months. The one- and two-year STFS rates were 62% (95%CI: 40-95%) and 54% (95%CI: 33-89%), respectively (Figure 2). The median OS was 34 months. The OS rates at one- and two-years were 71% (CI95% 52-96) (Figure 3). At the time of analysis, 13 deaths were identified, with one non-cancer-related death.

Adverse events

Among the entire cohort, no side effects were reported from SBRT, namely pulmonary or oesophageal toxicity. One patient developed a grade 1 cough during treatment, but was diagnosed with COVID-19, and this did not impede the continuation of treatment.

## Discussion

In the present cohort of 17 patients, we evaluated the efficacy of SBRT for the treatment of lung metastases from sarcoma, with a particular emphasis on the role of SBRT in delaying the initiation or modification of systemic therapy.

Prior studies have demonstrated the evolving role of radiotherapy in sarcoma management, particularly considering advancements in SBRT [11-18]. Though traditionally considered a second-line option due to the radioresistant nature of sarcomas, the ability of SBRT to deliver higher biologically effective doses has shifted this paradigm. Multiple studies highlight the capacity of SBRT to achieve remarkable local control rates, ranging from 83% to 96% at two years, with notable survival outcomes.

Frakulli et al. (2015) showed 85.9% two-year local control and 66.4% two-year OS in 24 patients with 68 metastases, while Baumann et al. (2016) reported 86% local control at two years, and 43% two-year OS [11-12]. Lindsay et al. (2018) achieved a 95% two-year local control rate for pulmonary metastases from sarcoma, with only six of 117 treated nodules showing post-treatment growth, and overall survival was 82% at two years and 50% at five years [13]. In a prospective series, Gutkin et al. (2023) showed a two-year local control rate of 96% and overall survival at two years of 74% and similarly, Farooqi et al. (2023) demonstrated a two-year local control rate of 83% and the median overall survival was 47 months [14,15]. In the largest retrospective series to date by Lebow et al. (2023), 66 patients treated with SBRT to 95 pulmonary sarcoma metastases, the 24-month cumulative incidence of local failure was only 7.4%, indicating high local control [16]. The median overall survival was 23 months, with a 24-month survival rate of 49% [16].

In addition to its efficacy in local disease control, SBRT has shown the potential to delay the initiation of systemic therapy, as reported in studies by Loi et al. (2018) and Asha et al. (2023) [17,18]. Loi et al. demonstrated that 56% of patients remained free of systemic therapy at two years, while Asha et al. reported that 85% of patients had not initiated systemic therapy one year after SBRT [17,18]. These findings underscore the role of SBRT as a viable strategy for providing therapeutic pauses or deferring the initiation of systemic treatments, particularly in patients with limited systemic options or those at risk of experiencing significant toxicities [17,18].

Our findings further support these observations, with a two-year LF after SBRT of 45.3% and allowing a median pause or delaying the initiation of systemic therapy of 26 months. This delay is of utmost importance in the context of sarcomas, given the limited systemic therapeutic options and the associated side effects. Effective control of localized disease with SBRT may not only improve quality of life but also optimize overall treatment strategies by minimizing the reliance on systemic approaches. It is worth noticing that the LC achieved in our cohort was lower than reported in the literature, potentially due to a lower BED10 in some treatments and broader criteria for patient selection.

Some limitations must be acknowledged. As a non-randomized, retrospective study, there was selection and reporting bias. Additionally, the relatively small sample size limits the statistical power and generalizability of the findings, while the heterogeneity of sarcoma subtypes and the varying treatment regimens further complicate direct comparisons and the drawing of robust conclusions.

Finally, the local control obtained with SBRT underscores its potential as a primary treatment modality for lung metastases in sarcoma. The evidence from our cohort, together with existing literature, suggests that SBRT should be considered an integral component of the multidisciplinary management of sarcoma lung metastases, offering both high efficacy with an urgent need to better tailor treatment timelines to individual patient needs.

## Conclusions

In conclusion, our study corroborates SBRT as a promising strategy for managing lung metastases from sarcoma. A median systemic therapy-free survival of 26 months and over half of the patients remaining off systemic therapy at two years, shows the possibility to optimize disease management with minimal toxicity. Although local control rates were slightly lower than those reported in other series, likely due to variations in BED delivery and patient selection, SBRT remained effective. These findings underscore SBRT as a valuable local therapy option for metastatic sarcoma, warranting further research to refine treatment protocols, optimize dose strategies, and identify patient subgroups that would benefit most, ensuring its integration into multidisciplinary sarcoma care.
